# Negative Skeletal Effects of Locally Produced Adiponectin

**DOI:** 10.1371/journal.pone.0134290

**Published:** 2015-07-31

**Authors:** Marcia J. Abbott, Theresa M. Roth, Linh Ho, Liping Wang, Dylan O’Carroll, Robert A. Nissenson

**Affiliations:** 1 Endocrine Research Unit, VA Medical Center and Departments of Medicine and Physiology, University of California San Francisco, San Francisco, CA, United States of America; 2 Department of Health Sciences and Kinesiology, Crean College of Health and Behavioral Sciences, Chapman University, Orange, CA, United States of America; Institut Albert Bonniot-INSERMU823, FRANCE

## Abstract

Epidemiological studies show that high circulating levels of adiponectin are associated with low bone mineral density. The effect of adiponectin on skeletal homeostasis, on osteoblasts in particular, remains controversial. We investigated this issue using mice with adipocyte-specific over-expression of adiponectin (AdTg). MicroCT and histomorphometric analysis revealed decreases (15%) in fractional bone volume in AdTg mice at the proximal tibia with no changes at the distal femur. Cortical bone thickness at mid-shafts of the tibia and at the tibiofibular junction was reduced (3–4%) in AdTg mice. Dynamic histomorphometry at the proximal tibia in AdTg mice revealed inhibition of bone formation. AdTg mice had increased numbers of adipocytes in close proximity to trabecular bone in the tibia, associated with increased adiponectin levels in tibial marrow. Treatment of BMSCs with adiponectin after initiation of osteoblastic differentiation resulted in reduced mineralized colony formation and reduced expression of mRNA of osteoblastic genes, osterix (70%), Runx2 (52%), alkaline phosphatase (72%), Col1 (74%), and osteocalcin (81%). Adiponectin treatment of differentiating osteoblasts increased expression of the osteoblast genes PPARγ (32%) and C/ebpα (55%) and increased adipocyte colony formation. These data suggest a model in which locally produced adiponectin plays a negative role in regulating skeletal homeostasis through inhibition of bone formation and by promoting an adipogenic phenotype.

## Introduction

Adipose tissue has been identified as a major regulator of metabolic homeostasis and is suggested to regulate bone mineral density (BMD)[1,2]. Excess adipose tissue has many detrimental effects, promoting the progression of heart disease, type 2 diabetes, metabolic disorders, and certain cancers. As such, adipose tissue depots in different locations play different regulatory roles in the maintenance of metabolic homeostasis and thus disease processes [3–5]. For instance, adipocytes located in the epigonadal regions are referred to as white adipocytes and their main function is to store energy in the form of triacylglycerides [3]. However, adipocytes residing in the bone marrow niche have yet to be fully characterized and are proposed to have different functions from adipocytes in the periphery [5,6]. In the bone marrow environment, mesenchymal stem cells (MSCs) give rise to both adipocytes and osteoblasts [3,7]. Additionally, there is growing evidence that competition for MSC commitment to either the adipocyte or osteoblast lineage exists [7–9]. In line with this notion, multiple studies have shown an inverse relationship between marrow adipocyte content and BMD in humans [10,11].

It has come to light, in recent years, that adipokines play a major role in regulating skeletal homeostasis [12–14]. Adiponectin has repeatedly been shown to be negatively associated with bone mass in humans [1,9,15,16]. However, unravelling a clear mechanistic role for adiponectin in the regulation of skeletal homeostasis has remained elusive [17–20]. Recently, it has been shown that adiponectin may exert its effects via both central and local mechanisms to regulate skeletal homeostasis with opposing outcomes [13]. Conversely, others have shown that genetically knocking out adiponectin results in decreases in trabecular bone formation while increasing marrow adiposity [21]. Further, there is evidence that adiponectin may have a protective role on bone formation in ovariectomized rabbits and mice [22,23]. Finally, marrow adipose tissue has been identified as a large contributor to circulating adiponectin levels [24]. Thus, the local adiponectin content, in the bone marrow, may be responsible for the negative skeletal phenotype in the adipocyte rich tibia. Overall, there has yet to be concordance or a clear regulatory role of adiponectin in the regulation of skeletal homeostasis. Taken together, the direct mechanisms by which adiponectin regulates bone formation and skeletal homeostasis has yet to be fully elucidated. The purpose of this study was to determine if increased local production of adiponectin, by adipocytes, elicits a site-specific effect on bone formation *in vivo* through a direct action on osteoblast lineage cells.

## Materials and Methods

### Animals

Animals were maintained on a normal chow diet (Prolab RMH 3000; Purina-Mills, St Louis, Missouri, USA) containing 26% protein, 14% fat, and 60% carbohydrate by calorie count; given free access to tap water; and housed in a room with a 12-hour light, 12-hour dark cycle and an ambient temperature of 22°C. All protocols were approved by the Animal Use Committee of the San Francisco Veterans Affairs Medical Center. Analyses were performed with transgenic mice (AdTg) that were kindly provided by Dr. Philipp E. Scherer, University of Texas Southwestern Medical Center [25]. The region encoding amino acids 57–95, containing 13 collagen repeats, was deleted using site-directed mutagenesis. The 5.4-kb aP2 enhancer/promoter sequence (provided by Bruce M. Spiegelman, Dana-Farber Cancer Institute, Boston, MA) was introduced into the MCS region upstream of the cDNA. In addition, the simian virus 40 splice and polyadenylase sequences were introduced into the MCS region downstream of the cDNA. For expression of the full-length protein, the complete open reading frame was inserted into the MCS of pCB7 (gift from J. Casanova, Massachusetts General Hospital, Boston, MA). The details of the generation of the AdTg mice have been previously described and used previously for successful production of transgenic mice, by mating AdTg males with FVB (WT) females [25]. Male and female mice at 12 weeks were characterized. Age- and sex-matched WT littermate mice were used as controls.

### Microcomputed Tomography (μCT)

Left femurs and tibiae were isolated and cleaned of adherent tissue. Before **μ**CT analysis, bones were fixed for 1–2 days in 10% phosphate buffered formalin (NBF; Fisher Scientific, Pittsburgh, Pennsylvania, USA) and stored in 70% ethanol for **μ**CT scanning. The femurs were imaged using a vivaCT-40 CT system (Scanco Medical AG, Bruttisellen, Switzerland). Imaging of cancellous bone was carried out at the distal metaphysic femur and proximal metaphysic tibia, and imaging of diaphyseal cortical bone was carried out at the tibiofibular junction (TFJ), mid-shaft of the tibia, and mid-shaft of the femur. All images were obtained using an x-ray energy of 55 kV with a voxel size of 10.5m on each side and integration time of 1000 msec. The cancellous region of interest was at a distance of 0.30–1.35 mm from the primary spongiosa. Quantitative assessment of diaphyseal cortex was conducted using data from 40 slices (0.42 mm) at the mid-femur. Cortical bone was assessed using a global thresholding protocol with segmentation values of 0.8/1/365.

### Static and Dynamic Histomorphometry

After **μ**CT analysis, the undecalcified bone samples were embedded in methyl methacrylate (MMA) and sectioned with Jung 2065 and 2165 microtomes (Leica, Bannockburn, Illinois, USA). Sectioned bones were processed for Von Kossa (VK) staining and tartrate resistant acid phosphatase (TRAP) staining as previously described [12,26]. Additional bones were decalcified and embedded in paraffin and Hemotoxylin and Eosin (H & E) staining procedures carried out. For dynamic histomorphometry, mice were injected with 20 mg/kg of calcein (Sigma-Aldrich, St Louis, Missouri, USA) 21 and 7 days before euthanasia and with 15 mg/kg of demeclocycline (Sigma-Aldrich, St Louis, Missouri, USA) 2 days before euthanasia. Mice were euthanized at 12 weeks, and femurs and tibia were isolated, fixed in neutral buffered formalin (NBF), and stored in 70% ethanol. After CT analysis, the undecalcified bone samples were embedded in MMA. Assessment of cancellous bone was performed on 10 **μ**m longitudinal sections from the left femur and the left tibia. Assessment of cortical bone was performed on 10 **μ**m transverse sections at the TFJ region. Mosaic-tiled images were acquired at 20X with a Zeiss Axioplan Imager M1 microscope (Carl Zeiss MicroImaging, Thornwood, New York, USA) fitted with a motorized stage. The tiled images were stitched and converted to a single image using the Axiovision software (Carl Zeiss MicroImaging, Thornwood, New York, USA) before blinded analyses were performed using image-analysis software (Bioquant Image Analysis Corp, Nashville, Tennessee, USA). Cancellous bone was assessed in the region 50 **μ**m from the lowest point on the growth plate, extending 1 mm down the metaphysis. The dynamic indices of bone formation that were measured include mineralizing surface per bone surface (MS/BS), mineral apposition rate (MAR), and surface-based bone-formation rate (BFR). Adipocytes were assessed in a region extending 500 **μ**mm from the growth plate on the lateral side of the tibia. To assess proximity of adipocytes to trabeculi, adipocytes less than or equal to 10 **μ**m to the trabecular bone were counted using ImageJ (National Institutes of Health, Bethesda, Maryland, USA) distance measurement tools.

### Serum Chemistry

Blood was collected from mice at the time of euthanasia and processed in MicroTainer serum separator tubes (BD Biosciences, San Jose, California, USA). Total and high molecular weight (HMW) adiponectin (mouse ELISA kit; Alpco Diagnostics) were analyzed. Plasma glucose levels were assessed using a glucometer.

### RNA extraction and RT-qPCR

Tissue samples were isolated and kept frozen in liquid nitrogen until processing. Before freezing, epiphyses were removed, and bone marrow was flushed from tibial bone samples. Frozen tissues were pulverized using a biopulverizer (Biospec Products, Inc, Bartlesville, Oklahoma), followed by RNA extraction using RNA STAT60 (Tel-Test, Inc, Friendswood, Texas) and subsequent purification using Micro-to-Midi Total RNA Purification kit (Invitrogen, Carlsbad, California, USA). cDNA was synthesized using TaqMan Reverse Transcription reagents (Applied Biosystems, Inc, Foster City, California, USA) and random hexamer primers according to the recommendations of the manufacturer. Gene amplification was measured with SYBR Green using the ABI Prism 7300 real-time thermocycler (Applied Biosystems, Inc, Foster City, California, USA). Analysis was carried out using the SDS software supplied with the thermocycler.

### Osteoblast Differentiation

A previously described procedure [12,27] was used to harvest bone marrow stromal cells (BMSCs) from WT and AdTg mice. The marrow was flushed from the long bones with primary culture media (PCM) consisting of α-modification of Eagle’s medium, supplemented with 10% fetal bovine serum (FBS), 100 U/mL penicillin, 100 g/mL streptomycin 0.25 μg/ml fungizone (Life Technologies, Carlsbad, CA, USA) from 12 week old male mice. The cells were collected in PCM and plated onto 6 well plates at a density 1 x 10^6^ cells/well for osteoblastic differentiation. After primary BMSCs had been maintained in primary medium for five days, the medium was aspirated and replaced with secondary osteogenic differentiation medium (primary medium containing 50 μg/ml ascorbic acid and 3 mM β-glycerolphosphate) to initiate osteoblast differentiation. For adiponectin treatment, differentiating BMSCs, isolated from WT mice, were treated with either 2.5, 5, or 10 μg/ml of adiponectin (R&D, Minneapolis, MN, USA) from day 14–28 of the differentiation protocol for assessment of osteoblastogenesis. PCM was changed three times each week and fresh adiponectin was added to the PCM at the specified concentration. Additionally, BMSCs were isolated from WT mice and treated with recombinant adiponectin (2.5 μg/ml; R&D Systems Inc, Minneapolis, Minnesota, USA) during the first 5 days of the osteoblastogenesis protocol. It has been shown that some commercially available recombinant adiponectin contains contaminating endotoxins [28] and to inhibit possible endotoxins polymyxin B (Sigma-Aldrich, St Louis, Missouri, USA) was added to the culture medium. At day 28, cells were fixed in NBF. VK and alkaline phosphatase staining was performed as previously described to assess osteoblastogenesis at the end of the 28 day differentiation protocol [29]. Briefly, the presence of alkaline phosphatase activity was assessed using a Leukocyte Alkaline Phosphatase kit (Sigma-Aldrich, St Louis, Missouri, USA). Two percent silver nitrate (Sigma-Aldrich, St Louis, Missouri, USA) solution was added to the cell culture dishes and UV-crosslinked for 10 min. Staining solution was aspirated and stained dishes were rinsed twice with distilled water for assessing mineralized nodules. For quantification of the colony formation and mineralized nodules, stained cultures were scanned and analyzed using the colony counter plug-in for Image J software. To assess differentiating osteoblasts for an adipogenic phenotype, isolated BMSCs from WT mice were treated with adiponectin from day 14–28 of osteoblast differentiation and fixed as aforementioned. Prior to Oil Red O or Nile Red staining cells were treated with or without 3 mM oleate for 12 hours in order to induce lipid accumulation and visualize adipocyte like cells. Cells were stained with Oil Red O for 1 hour and rinsed several times with distilled water to remove any excess stain.

### Adipocyte Differentiation

The marrow was flushed from the long bones of 12 week old WT and AdTg mice and isolated BMSCs were cultured in PCM. The cells were plated onto 6 well plates at 10 x 10^6^ cells/well for adipogenic differentiation. The PCM was replaced with secondary adipogenic differentiation medium (addition of 1 μM rosiglitazone, 1 μM dexamethasone, 5 μg/mL insulin, and 500 μM 3-isobutyl-1-methylxanthine) at day 10 of culture. After 2 days in secondary adipogenic medium, this medium was replaced with PCM containing 5 μg/mL insulin for two additional days, and then cells were maintained in PCM until day 19. At day 19, cells were fixed in NBF. After fixation, cells were stained in Oil Red O (Sigma-Aldrich, St Louis, Missouri, USA) to assess adipogenesis. Cells were rinsed several times with distilled water to remove excess stain. Stained cultures were air dried, scanned, and analyzed using the colony counter plug-in ImageJ (National Institutes of Health, Bethesda, Maryland, USA) software for quantifying the lipid containing colonies. For quantification of total staining on oleate treated cells the Oil Red O was extracted from the plate with 75% ethanol, analyzed at an absorbance at 490 nm, and normalized to the vehicle control. For Nile Red staining cells were fixed as aforementioned and a commercially available (Santa Cruz Biotechnology, Santa Cruz, California, USA) neutral lipid stain was added to the cells and counter stained with DAPI to visualize individual nuclei.

### Immunohistochemistry

BMSCs from 12-week-old mice harboring a 2.3 Col1-GFP transgene were flushed and plated on coverslips in 24-well plates in PCM. Cells were switched to osteogenic media days 7–14, then media was replaced with secondary adipogenic media and treated with or without adiponectin (2.5 μg/ml) from day 14–16. Cells were cultured for another 2 days in PCM with 5 μg/ml insulin and treated with or without adiponectin (2.5 μg/ml), and then an additional 4 days in standard PCM with or without adiponectin (2.5 μg/ml). At day 23, cells were fixed with NBF, washed with tris-buffered saline (TBS), and permeabilized with 0.05% digitonin. Cells were blocked with 2% bovine serum albumin and incubated with antibodies against fatty acid binding protein 4 (FABP4; 1:500) antibody (Cayman Chemical, Ann Arbor, MI, USA), overnight before staining with Alexa Fluor 594-coupled secondary antibody (Life Technologies, Carlsbad, CA, USA). Cells were then washed and mounted with DAPI to visualize nuclei. Images were taken by using a Spinning Disk Confocal of UCSF NiKon Imaging Center (NIC) at 60X x 1.4 magnification

### Statistical analysis

All data are presented as mean ± SEM and each experiment was replicated at least three times with at least 3 internal replicates. Statistical significance was ascertained by comparison between AdTg mice and sex-matched WT controls or vehicle control cells and cells treated with adiponectin using two tailed Student’s t test. Statistical significance was set as *P*<0.05.

## Results and Discussion

### General Phenotype of AdTg Mice

There were no significant differences observed in body weight and blood glucose in AdTg mice when compared to sex matched WT mice (Table 1). Similar to previously published results, serum adiponectin levels were 59% and 71% higher in AdTg female and male mice, respectively, when compared to WT sex matched mice [25]. Tibias were 4.4% shorter in female AdTg mice and 4.8% shorter in male AdTg mice when compared to WT sex matched mice (*P*<0.01). There were no significant differences in femur lengths between WT and AdTg mice in either sex (Table 1).

**Table 1:** Phenotypic characteristics of 3 month old WT and AdTg mice.

	Females		Males	
	WT	AdTg	WT	AdTg
Body Weight (g)	22.5±0.8	22.2±0.5	28.0±0.8	27.4±0.6
Femur Length (mm)	14.9±0.1	14.7±0.3	15.2±0.2	15.0±0.1
Tibia Length (mm)	18.1±0.2	17.3±0.2**	18.6±0.2	17.7±0.1*
Blood Glucose (mgdL)	131.3±16.2	137.3±11.8	142.7±15.3	133.0±13.9
Adiponectin (ug/ml)	38.5±3.8	61.1±2.1*	25.3±2.8	43.8±6.2*
HMW Adiponectin (ug/ml)	7.5±0.3	10.4±0.1**	6.9±0.2	9.1±0.1

WT, wild type; AdTG, adiponectin transgenic mice; HMW, high molecular weight

The values are expressed as the mean ± SEM (n = 4–8). *P*<0.05*; *P*<0.01** significantly different from WT.

### Skeletal Phenotype in AdTg Mice

**Fig 1:**
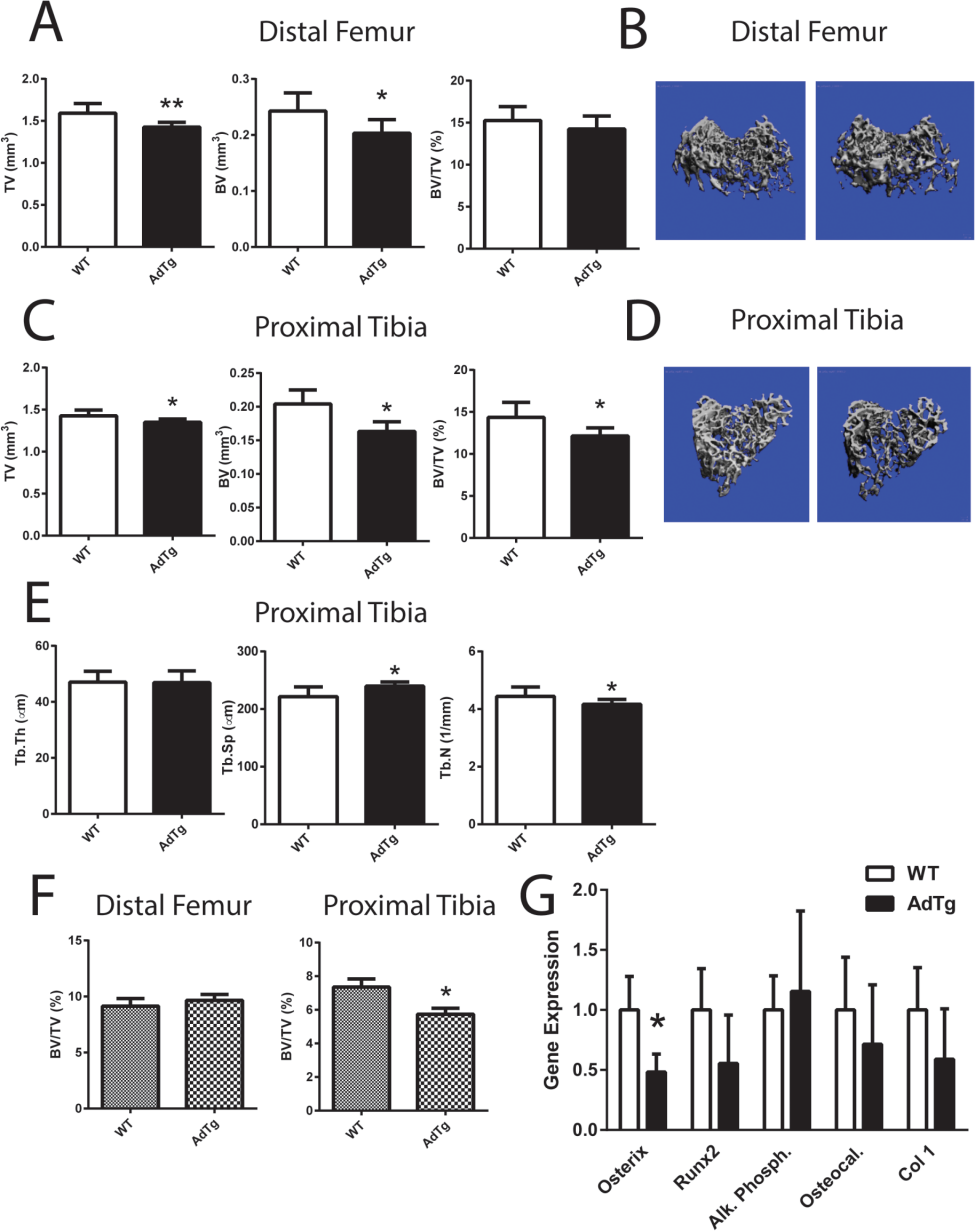
Cancellous bone measurements. Bone tissue volume refers to the entire volume of bone scanned (TV), bone volume refers to the volume of mineralized bone within the scanned region (BV) and fractional bone volume referring to the fraction of mineralized bone relative to the total bone volume (BV/TV) was assessed by A) μCT and B) three-dimensional reconstruction μCT renderings at the distal femur and C) TV, BV, and BV/TV at the proximal tibia D) three-dimensional reconstruction images at the proximal tibia D). E) Trabecular thickness (Tb.Th), spacing (Tb.Sp.), and number (Tb.N) was assessed at the proximal tibia by μCT analysis. F) Histomorphometric analysis of distal femur and proximal tibia using Bioquant Software. G) Expression level of osbteoblast marker genes: Osterix, Runx2, Alkaline Phosphatase (Al. Phosph.), Osteocalcin (Osteocal.) and Collagen Type I (Col 1). All expression data were obtained by RT-qPCR analysis of RNA and calculated based on the ddCt method with GAPDH as the reference gene and WT as the control. All data are shown as mean ± SEM from 12 week old female mice (n = 5–8). Statistical significance * *P*<0.05, ** *P*<0.01, WT compared with AdTg bones.

To assess cancellous bone mass in AdTg mice, distal femurs and proximal tibia from male and female mice were examined using both μCT and histomorphometry. There were no measured significant differences in any cancellous bone parameters in both the distal femur and proximal tibia in male AdTg mice when compared to WT sex matched mice (S1 Fig). However, tissue volume (TV) and bone volume (BV) were significantly lower in female distal femurs in AdTg mice (*P*<0.05, Fig 1A and 1B). There were no significant differences in overall fractional bone volume (BV/TV; Fig 1A and 1B). The cancellous TV, BV, and BV/TV at the proximal tibia were significantly lower in AdTg females (*P*<0.05, Fig 1C and 1D). The decrease in BV/TV was associated with a decrease in trabecular number and an increase in trabecular spacing in AdTg female mice (*P*<0.05, Fig 1C–1E). Histomorphometry analysis concurred with the μCT data (Fig 1F and S1 Fig). Bone marrow stromal cells (BMSCs) isolated from the tibia from WT and AdTg mice exhibited no alterations in their ability to differentiate into osteoblastic cells, when assessed by alkaline phosphatase and VK staining (S1 Fig). Gene expression of key osteoblastic markers (osterix, Runt-related transcription factor 2 (Runx2), alkaline phosphatase, osteocalcin, and collagen type 1) within the isolated tibia were measured and osterix was the only marker shown to be significantly lower in AdTg mice when compared to WT mice (*P*<0.05, Fig 1G).

**Fig 2:**
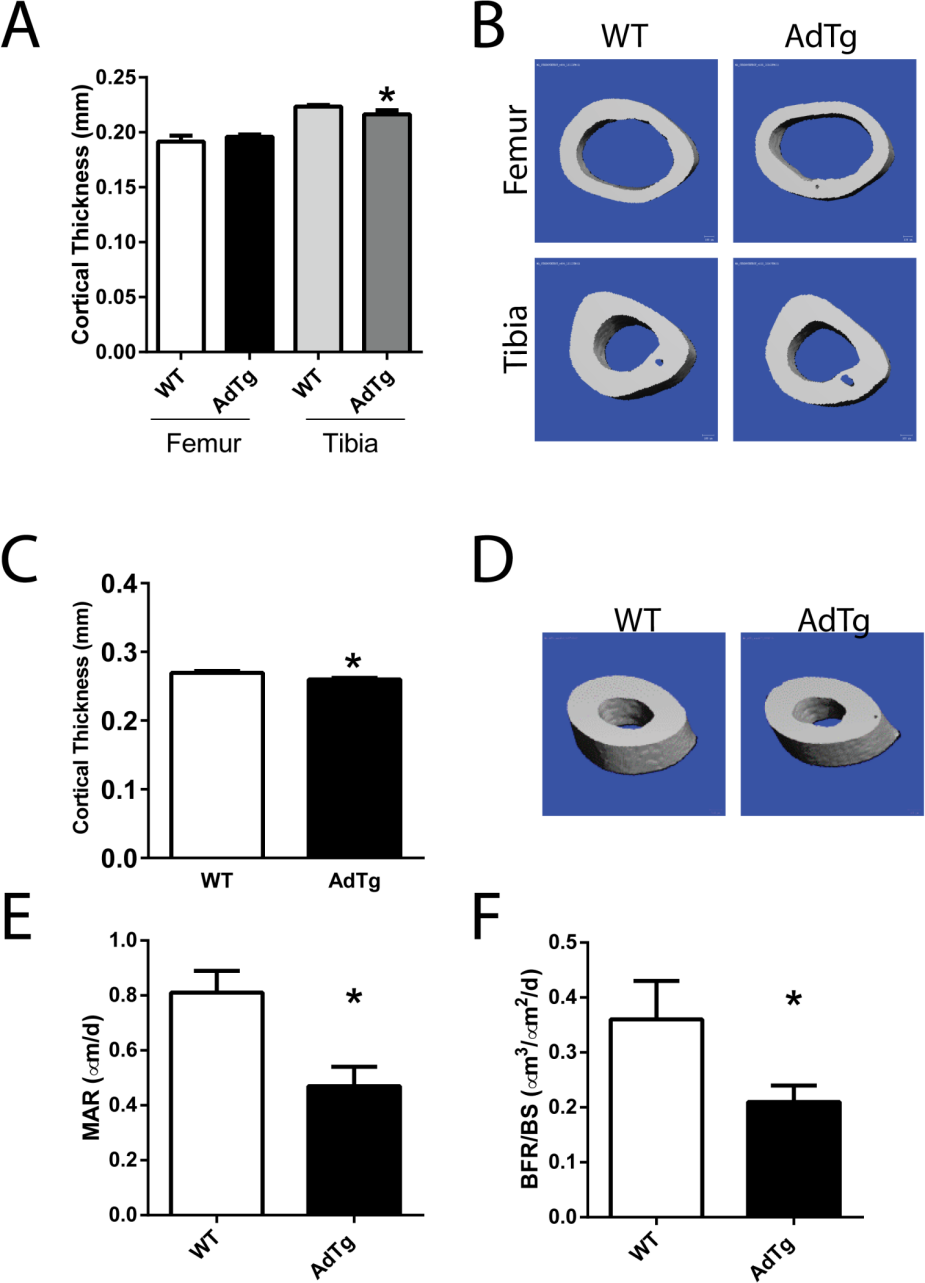
Cortical bone parameters of the midshaft of femurs and tibia and histomorphometry. A) μCT and B) three-dimensional reconstruction μCT renderings. C) Cortical thickness assessed at the Tibiofibular junction (TFJ) and D) three-dimensional reconstruction μCT renderings of the (TFJ). E) Mineral apposition rates and F) bone formation rates measured by dynamic histomorphometry at the proximal tibia. All data are shown as mean ± SEM from 12 week old female mice (n = 5–8). Statistical significance * *P*<0.05, WT compared with AdTg bones.

Cortical bone of AdTg mice was assessed by μCT at the femoral and tibia mid-shafts and at the tibiofibular junction (TFJ; Fig 2). Cortical thickness was significantly smaller at the mid-shaft of the tibia in female AdTg mice when compared to WT mice (*P*<0.05, Fig 2A and 2B). In female AdTg mice cortical thickness at the TFJ was significantly smaller than in WT mice (*P*<0.05, Fig 2C and 2D). Cortical thickness was not significantly different in male AdTg femurs, tibia, or TFJ when compared to WT mice (S2 Fig). Dynamic histomorphometry was performed in the proximal tibia of female WT and AdTg mice. Female AdTg mice displayed a decrease in mineral apposition rate and bone formation rates while no differences in osteoblast recruitment (data not shown) at the proximal tibia indicating impairment of osteoblast function (*P*<0.05, Fig 2E and 2F). Osteoclast numbers per bone surface were not different between the WT and AdTg mice (S3 Fig). Static histomorphometry analysis revealed a decrease in osteoclast surface in the proximal tibia of female AdTg mice (*P*<0.5, S3 Fig).

### Adipogenic Phenotype in AdTg Mice

**Fig 3:**
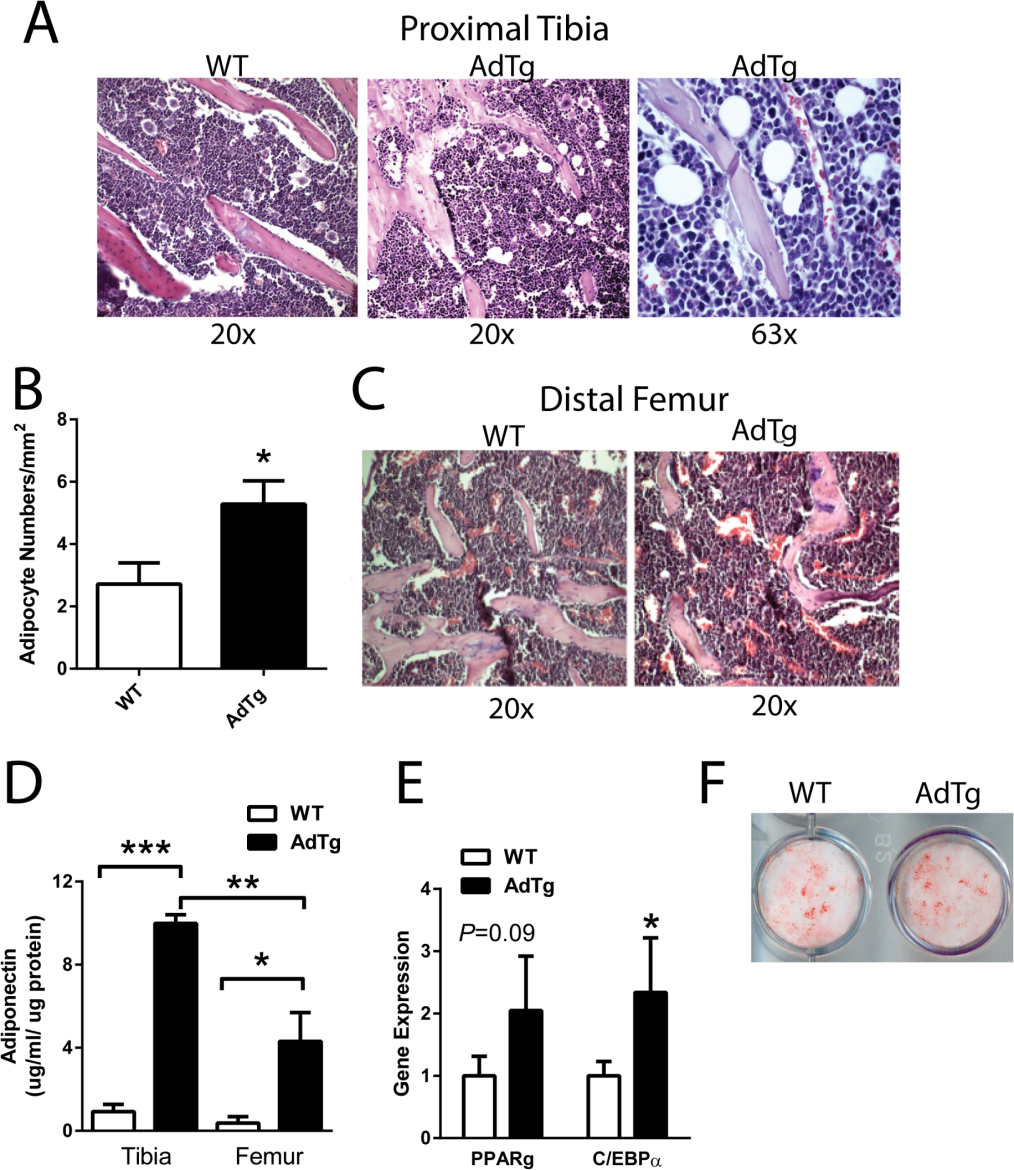
Effects of adiponectin on adipogenesis. Hemotoxylin and Eosin (H & E) staining of A) proximal tibia and B) number of adipocytes measured to be ≤ 10 **μ**m from trabeculi. C) H & E staining of distal femur. D) Adiponectin concentration measured in the marrow from tibia and femurs. E) Expression level of the adipocyte marker genes: peroxisome proliferator-activated receptor gamma (PPARγ) and CCAAT/enhancer-binding protein alpha (C/ebpα). Assessment of adipogenesis F) by Oil Red O staining, from BMSCs isolated from the tibia of WT and AdTg mice. All expression data were obtained by RT-qPCR analysis of RNA and calculated based on the ddCt method with GAPDH as the reference gene and WT as the control. All data are shown as mean ± SEM from 12 week old female mice (n = 3–6). Statistical significance * *P*<0.05, WT compared with AdTg bones.

Upon histological examination, AdTg female mice exhibited increased numbers of adipocytes in close proximity to trabeculi (<10 **μ**m) in the tibia when compared to WT mice (Fig 3A and 3B). Similar to the lack of trabecular phenotype in the distal femurs, these findings were not present in the distal femur (Fig 3C). Consistent with a proposed local regulation of adiponectin on skeletal homeostasis, marrow adiponectin content was higher in the tibia compared to the femur (P<0.05, Fig 3D). The expression of the adipogenic marker genes CCAAT/enhancer-binding protein alpha (C/ebpα) and PPARγ were elevated in the tibia, flushed of marrow, of AdTg mice although only C/ebpα reached statistical significance (*P*<0.05, Fig 3E). Interestingly, BMSCs isolated from the tibia of AdTg mice displayed no impairment in their ability to differentiate into adipocytes (Fig 3F).

### Effects of Adiponectin on Osteoblastogenesis *In Vitro*

**Fig 4:**
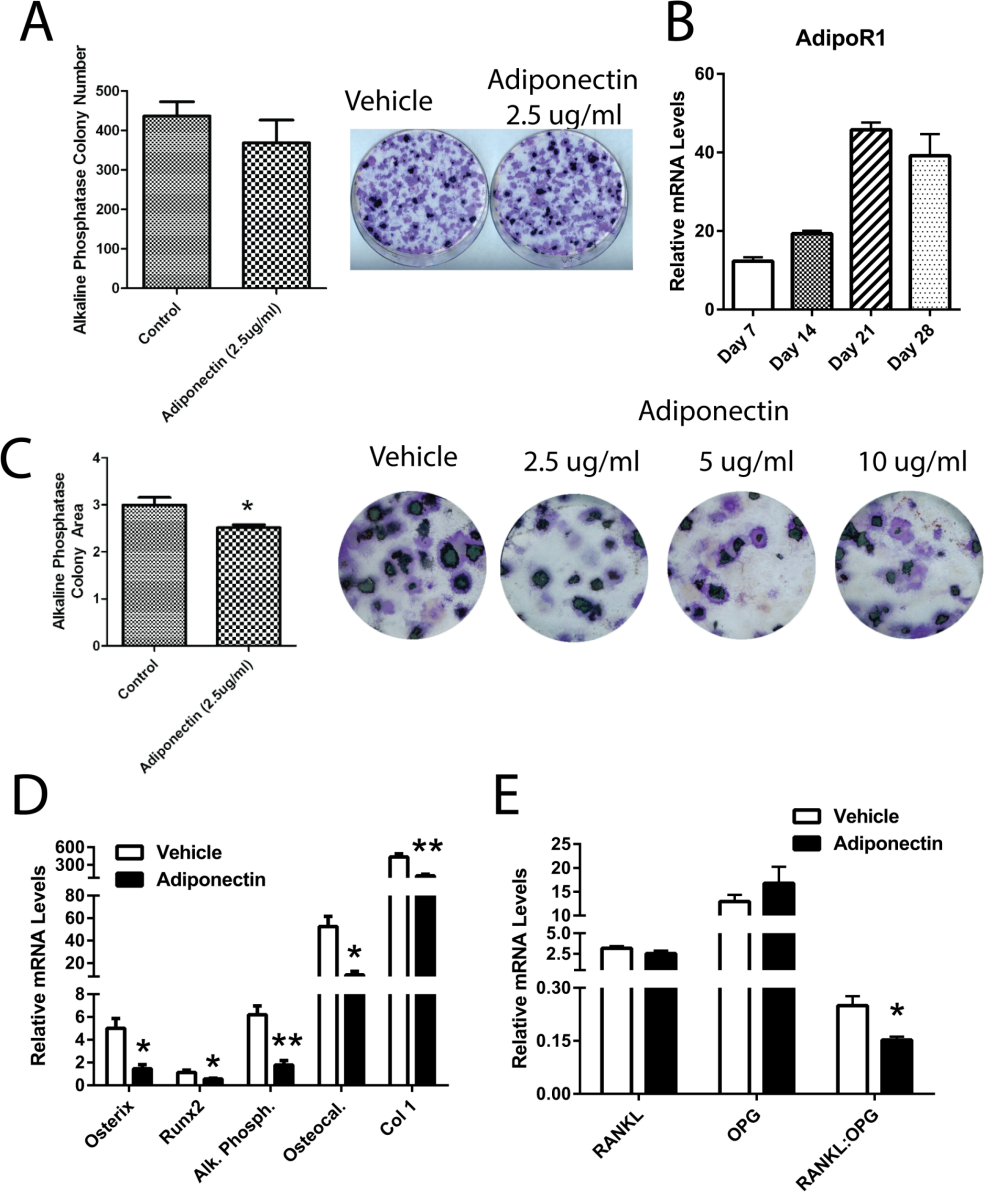
Adiponectin regulates osteoblastogenesis in bone marrow stromal cells. A) Effect of adiponectin treatment (2.5 μg/ml) from day 0–5 on osteoblast differentiation from BMSCs as assessed by Von Kossa (VK) and alkaline phosphatase staining. B) mRNA expression levels of the adiponectin receptor 1 (AdipoR1) throughout 28 days of osteoblastic differentiation. C) Effect of adiponectin treatment (2.5, 5, and 10 μg/ml) from day 14–28 on osteoblast differentiation from BMSCs as assessed by VK and alkaline phosphatase staining and on D) expression levels of osteoblast markers: osterix, Runx2, alkaline phosphatase (Al. Phosph.), osteocalcin (Osteocal.) and collagen type I (Col 1). E) Expression levels of RANKL:OPG. Colony numbers were calculated using Image J Software. All expression data were obtained by RT-qPCR analysis of RNA and GAPDH as the relative control. All data are shown as mean ± SEM from BMSCs isolated from 12 week old WT mice (n = 3–7). Statistical significance * *P*<0.05.

Adiponectin treatment in the first 5 days of osteoblastogenesis, in BMSCs isolated from WT mice, had no effect on the cells’ ability to differentiate into osteoblasts (Fig 4A). The level of expression of the adiponectin receptor 1 (AdipoR1) gene increased throughout differentiation of BMSCs towards mature osteoblastic cells (*P*<0.05, Fig 4B). Therefore we examined the effects of adiponectin on osteoblastic cells at later stages of differentiation. Treatment of differentiating osteoblastic cells with adiponectin from days 14–28, impaired their ability to fully differentiate as assessed by alkaline phosphatase activity and VK staining (*P*<0.05, Fig 4C). The ability of adiponectin to impair osteoblastogenesis was maximal at 2.5 ug/ml adiponectin (Fig 4C). The expression of genes associated with osteoblastogenesis (osterix, Runx2, alkaline phosphatase, osteocalcin, and collagen type I) was significantly lower in adiponectin treated osteoblastic cells when compared to control cells (*P*<0.05, Fig 4D). Additionally, the RANKL/OPG ratio was significantly lower in the cells treated with adiponectin when compared to the vehicle control cells (*P*<0.05, Fig 4E).

### Adiponectin Stimulates Mature Osteoblastic Cells Towards an Adipogenic Phenotype

**Fig 5:**
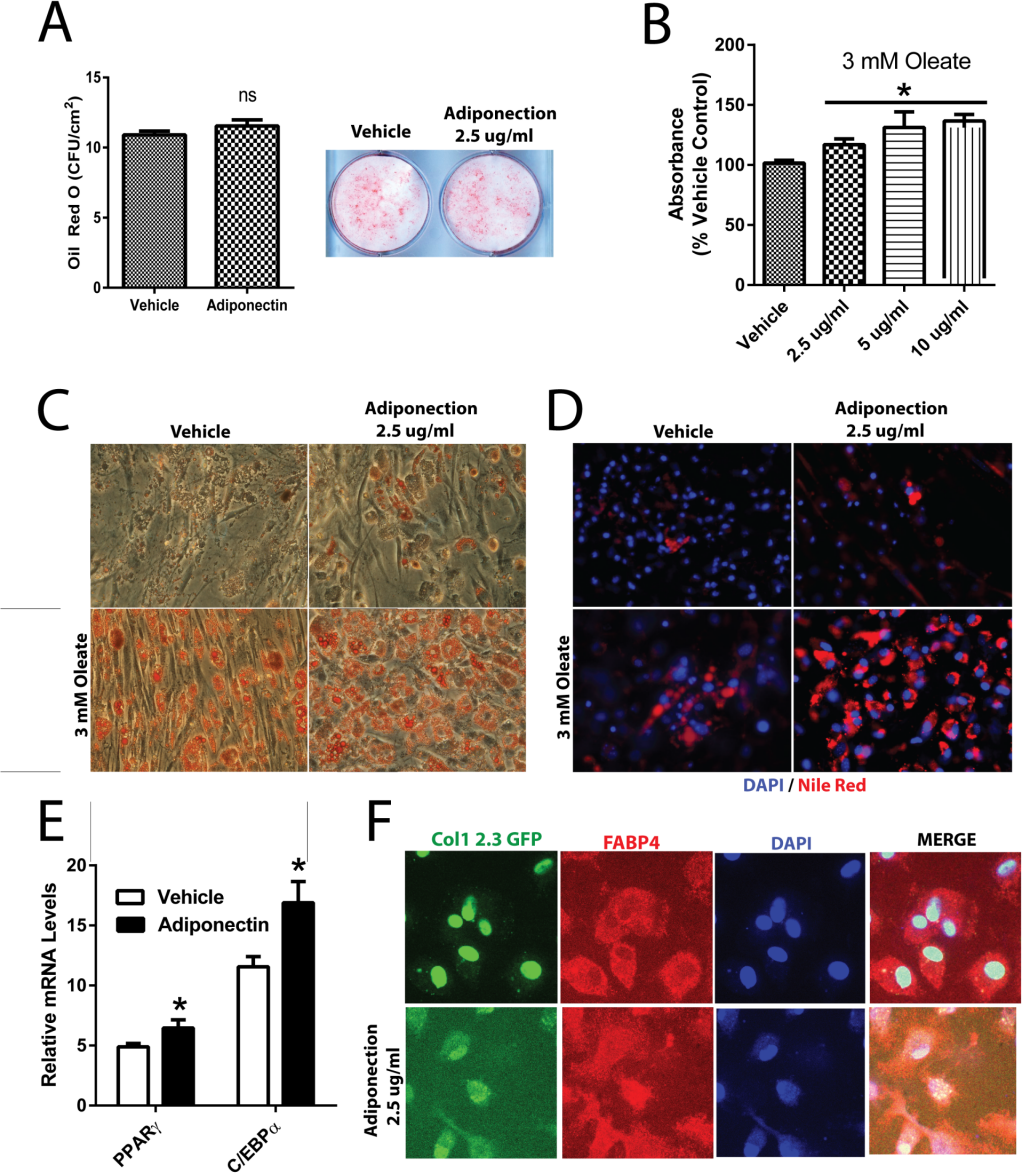
Effects of Adiponectin on adipogenesis. A) Effect of adiponectin treatment (2.5 μg/ml) from day 0–5 on adipogenic differentiation from BMSCs as assessed by Oil Red O staining. Effect of adiponectin treatment (2.5 μg/ml) from day 14–28 on adipogenesis as assessed by B) quantification and C) visualization of Oil Red O staining of adipocyte-like cell colonies and D) Nile Red staining with or without 3 mM oleate. Red staining represents lipid droplets and blue staining represents DAPI stained nuclei. E) Expression level of the adipocyte marker genes Pparg and C/ebp alpha. F) Co-localization studies of BMSCs isolated from Col-1 2.3 promoter driven GFP reporter mice treated with 2.5 μg/ml of adiponectin in adipogenic media from day 14–21 following osteoblastic differentiation for 7 days. Green staining represents osteoblast lineage cells, red staining indicates adipogenic marker FABP4, and blue staining indicates DAPI stained nuclei. Cells were visualized with a spinning disk confocal microscope. Colony numbers were calculated using Image J Software. All expression data were obtained by RT-qPCR analysis of RNA and GAPDH as the relative control. All data are shown as mean ± SEM from BMSCs isolated from 12 week old WT mice (n = 3–7). Statistical significance * *P*<0.05.

Treatment of BMSCs isolated from WT mice with adiponectin had no effect on the cells’ ability to differentiate towards adipocytes when adiponectin was added during the first 5 days of differentiation (Fig 5A). However, treatment of mature differentiating osteoblastic cells with adiponectin resulted in an increase in Oil Red O and Nile Red staining (*P*<0.05, Fig 5B–5D). The effect of adiponectin on Oil Red O staining was measured at the lowest dose of adiponectin (2.5 **μ**g/ml) (Fig 5B). When 3 mM oleate was added to the culture medium, to stimulate lipid droplet formation, the morphology of the osteoblastic cells shifted towards a more “adipocyte-like” cell in the presence of adiponectin from day 14–28 of differentiation (Fig 5C and 5D). In line with the increased lipid staining, mature osteoblastic cells treated with adiponectin resulted in an increase in mRNA levels of adipogenic markers PPARγ and C/ebpα (*P*<0.05, Fig 5D). Finally, when differentiating osteoblasts, verified by osteoblastic Col-1 2.3 promoter driven GFP expression, were redirected towards adipogenic cells, adiponectin enhanced the adipogenic phenotype, as assessed by FABP4 co-localization with the GFP positive cells (Fig 5F).

## Conclusions

We demonstrate here that in 3 month old female mice increased circulating adiponectin has negative effects on skeletal homeostasis, specifically in the proximal tibia without observed effects in the distal femur. Additionally, our results show that there is an increased number of adipocytes in close proximity to trabeculi in the tibia of the AdTg mice without alterations in adipocyte content in the distal femur. Taken together, these results indicate that the negative effects of adiponectin on trabecular bone are exerted in a location specific manner. Our data also show that adiponectin exerts its negative effects on differentiating osteoblasts rather than MSC progenitors. Importantly, our data indicate that adiponectin promotes a “redirection” of differentiated osteoblasts towards “adipocyte-like” cells *in vitro*, an effect that may contribute to the observed skeletal phenotype. Overall, it is clear, from the current study, that adiponectin plays an important negative role in the regulation of skeletal homeostasis.

The identification of cytokines secreted from the adipose tissue (adipokines), such as leptin and adiponectin, have led to the acceptance that adipose tissue has functions beyond fat storage. Indeed, we have previously shown that the adipokine apelin exerts negative effects on skeletal homeostasis [12]. More recently adiponectin has been proposed to be a regulator of skeletal homeostasis, beyond its regulatory role in modulating many metabolic processes, obesity, type 2 diabetes, and heart disease [9,15,30]. In humans, numerous studies have identified an inverse correlation between circulating adiponectin levels and BMD [1,9]. These findings might seem paradoxical in that adiponectin is viewed as a beneficial hormone for overall metabolic health. A similar situation exists with the diabetic drug class thiazolidinediones (TZDs) which activate PPARγ and adipogenesis [3,4,31]. Diabetic patients given TZDs achieve increases in insulin sensitivity and adipose tissue at the expense of BMD [4]. Based on these observations it is conceivable that adiponectin may act to increase insulin sensitivity while decreasing bone formation and ultimately favoring adipogenesis. A recent report suggests that adiponectin reduces BMD by a direct action on bone during growth but can also increase BMD during aging by an action on the CNS to inhibit sympathetic tone [13]. Further, adipocytes residing in the bone marrow niche have been identified as important endocrine regulators of adiponectin secretion [24]. Our data support the notion that the local environment plays an important role in determining the effects of adiponectin on bone formation. For instance, we show that increases in circulating adiponectin in AdTg mice had no obvious effects on the trabecular phenotype in the distal femur. Although, the marrow content within the AdTg mice showed an increase in adiponectin, the levels were not as high as in the tibia. It can be speculated that perhaps the decreased bone phenotype is dependent on the presence of endogenous adipocyte levels. However, the proximal tibia, which was exposed to higher local levels of adiponectin, displayed decreases in fractional bone volume and bone formation rates. We also provide evidence for a role of adiponectin in locally regulating bone resorption. In this study, we found that adiponectin significantly decreased the ratio of RANKL/OPG in the osteoblast cultures. The alterations in osteoclast number that are shown here, are also agreement with other reports of adiponectin negatively impacting osteoclastogenesis [32]. Collectively, the excessive endogenous adiponectin resulted in a low turnover type of bone loss in female mice, in which the decreased fractional osteoclast surface is mainly associated with a dramatic decrease of bone formation in the proximal tibia of the female AdTg mice. Although we did not observe a significant decrease of bone mass within either cortical or cancellous bone compartment in femoral bones, a previous study on the same mice has proven that high circulating adiponectin impairs femoral cortical bone mechanical properties [17]. Further, it has been suggested that adiponectin may affect bone biochemical properties as well [17].

Although adiponectin exerts positive effects in regulation of metabolic disorders, it appears to have negative effects on skeletal tissue [15,33]. Interestingly, our data show that there are increased adipocytes residing close to the trabeculi in the tibia but not in the distal femur of the AdTg mice. Further we measured greater adiponectin content in the marrow of the tibia when compared to that of the femur. In line with the observed adipogenic phenotype, we measured an increase in C/ebpα expression in the tibia. Therefore, it is likely that the local effects of adiponectin on trabecular bone formation are a result of direct action on neighboring osteoblasts. However, it is not clear if adiponectin action acts as a feed forward mechanism on adipogenesis or if it “redirects” osteoblasts towards an adipocyte phenotype.

Our *in vitro* data show that one action of adiponectin on osteoblasts may be to “redirect” or promote adipogenesis of differentiating osteoblasts towards “adipocyte-like” cells. However, due to the heterogeneity of the cultured cells, we cannot rule out the possibility that adiponectin may be acting on another population of cells in the culture conditions. Nevertheless, the notion of adiponectin action in promoting adipogenesis is in line with other reports that show that adiponectin may be a key factor in the balance between Runx2 and PPARγ gene expression [11,34]. Conversely, others have shown that adiponectin may act to stimulate Runx2 to promote osteogenesis [35]. On the other hand, it has recently been shown that adiponectin has the ability to promote hepatic stellate cells towards an adipogenic pathway [36]. In our studies, the morphology of the osteoblastic cells were altered and appeared similar to differentiating adipocytes when treated with adiponectin. Further, when stimulated with the addition of oleate, the cells treated with the adiponectin had a greater propensity to create lipid droplets which is a hallmark of adipocyte function and these findings were confirmed by increases in the adipogenic genes PPARγ and C/ebpα. Finally, it is clear that adiponectin acts to enhance an adipogenic phenotype on osteoblasts as evidenced by the co-localization of the bone specific marker, Col1, with the adipogenic marker FABP4. Indeed, we show that adiponectin acts to “redirect” differentiating osteoblasts towards adipocyte like cells. However, here we show no evidence for adiponectin regulation in stem cell determination since BMSCs isolated from AdTg mice were able to differentiate to osteoblasts or adipocytes similarly to BMSCs from WT mice. Further, the ability of BMSCs isolated from WT mice to differentiate to osteoblasts or adipocytes was unaffected by the addition of adiponectin during the first five days in culture. Overall, these findings suggest that adiponectin acts to “redirect” or “transdifferentiate” cells committed to the osteoblastic lineage towards the adipogenic lineage, which possibly accounted for the decreased bone formation in the female AdTg mice.

Although the data presented here show that adiponectin exerts negative effects on bone tissue, the mechanism remains to be determined. Adiponectin is known to signal through its receptors (Adipo R1 and Adipo R2) that have been shown to be present in osteoblasts [19,37]. The most widely accepted pathway for adiponectin signaling is through the master energy sensing enzyme AMP-activated protein kinase (AMPK) [37,38]. However, evidence for adiponectin signaling through AMPK action in osteoblasts, to regulate skeletal homeostasis, has yet to be confirmed [13]. Recently it has also been shown that Adipo R1 and Adipo R2, once activated by adiponectin, may act as ceramidases [39]. Therefore it is conceivable that the negative effects of adiponectin may be mediated by increases in metabolites resulting from ceramide breakdown, such as sphingosine-1-phosphate (S1P). S1P has been shown to act through its receptors which have been identified as G-protein coupled receptors (GPRCs) [40]. In particular S1P has been shown to exert its actions through Gi-coupled receptor signaling [40]. In previous studies, we have shown that activation of the Gi-protein signaling pathway in osteoblasts results in suppression of bone formation [12,41,42]. However, we (unpublished observations) and others have been unable to demonstrate an effect of adiponectin on S1P levels in osteoblasts [13]. Interestingly, it has been shown that global overexpression of Adipo R1 resulted in positive effects on skeletal homeostasis [43]. This is likely to be due to the actions of adiponectin on AdipoR1 in cells other than osteoblasts. Taken together, it is clear that the various genetic models used to study adiponectin have brought to light the complexity of adiponectin regulation on skeletal homeostasis and requires further examination.

In conclusion, the present study indicates that the adipokine adiponectin has overall negative effects on bone formation *in vivo* and *in vitro*. It can be concluded that the effects of adiponectin on skeletal homeostasis are dependent on either endocrine or paracrine actions. Importantly, the data indicate that adiponectin has direct negative effects on cells under osteoblastic culture conditions and may promote their “redirection” towards an adipogenic lineage. Overall, we show here that the negative effects of adiponectin on bone formation may be an important contributor to metabolic bone diseases.

## Supporting Information

S1 Fig Cancellous bone measurements in male mice. (TIF)

S2 Fig Cortical bone measurements in male mice. (TIF)

S3 Fig Adiponectin effect on osteoclastogenesis. (TIF)
