# Supplementary figures and images for: Negative Skeletal Effects of Locally Produced Adiponectin
Source: PLoS One. 2015 Jul 31;10(7):e0134290. doi: 10.1371/journal.pone.0134290 (PMC4521914; doi:10.1371/journal.pone.0134290)

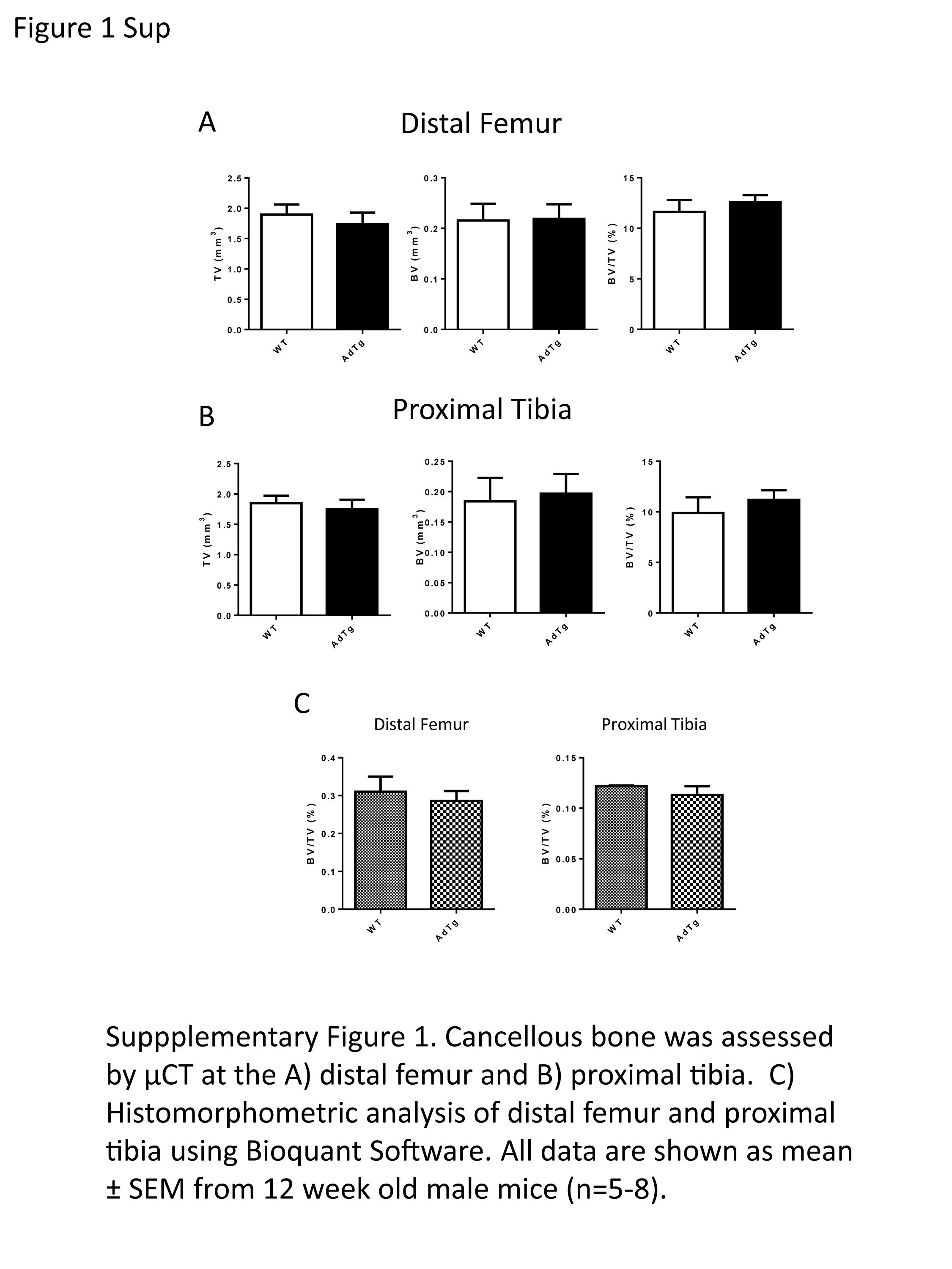

Supplement: S1 Fig — (TIF) [file pone.0134290.s001.tif]

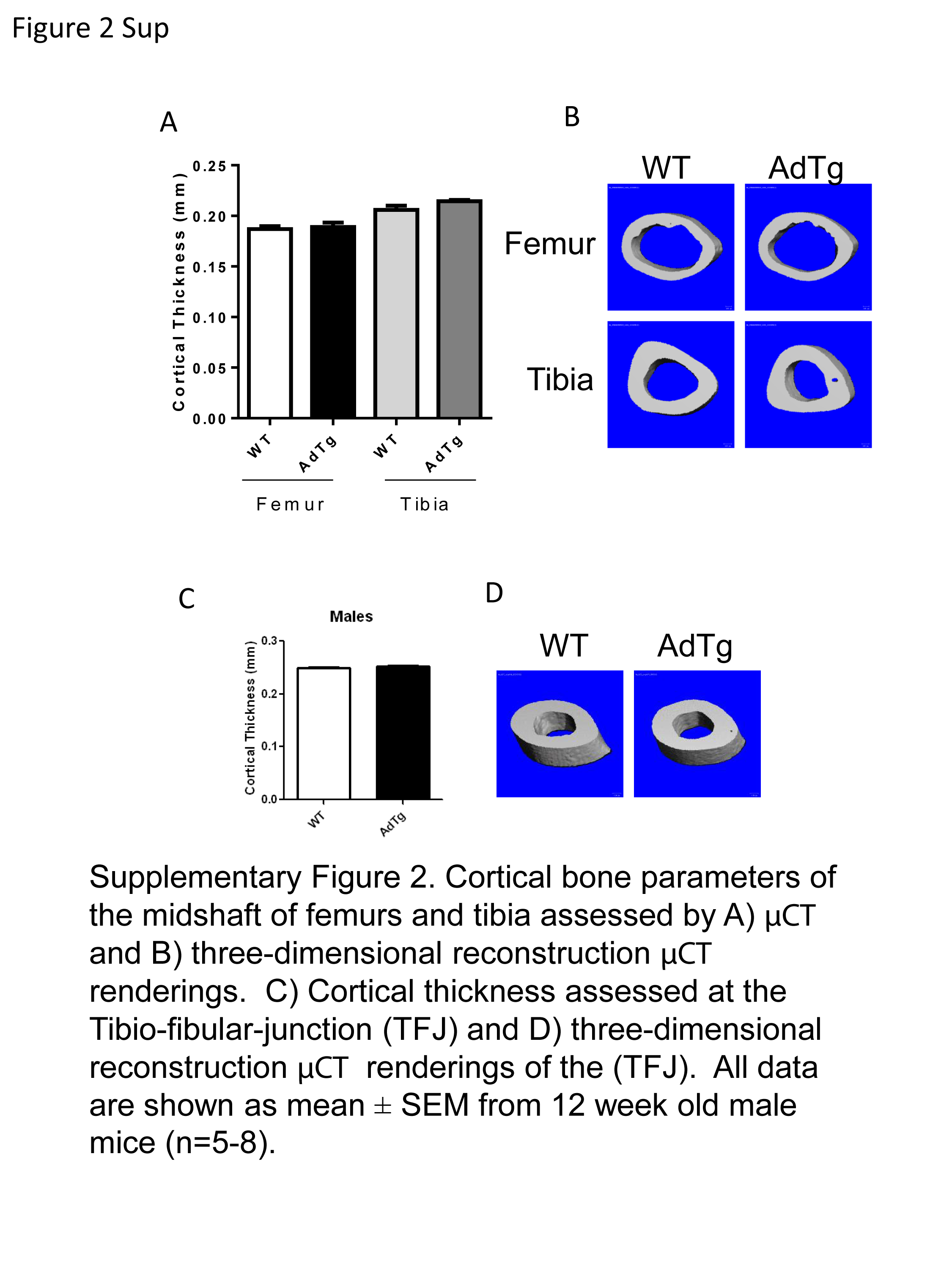

Supplement: S2 Fig — (TIF) [file pone.0134290.s002.tif]

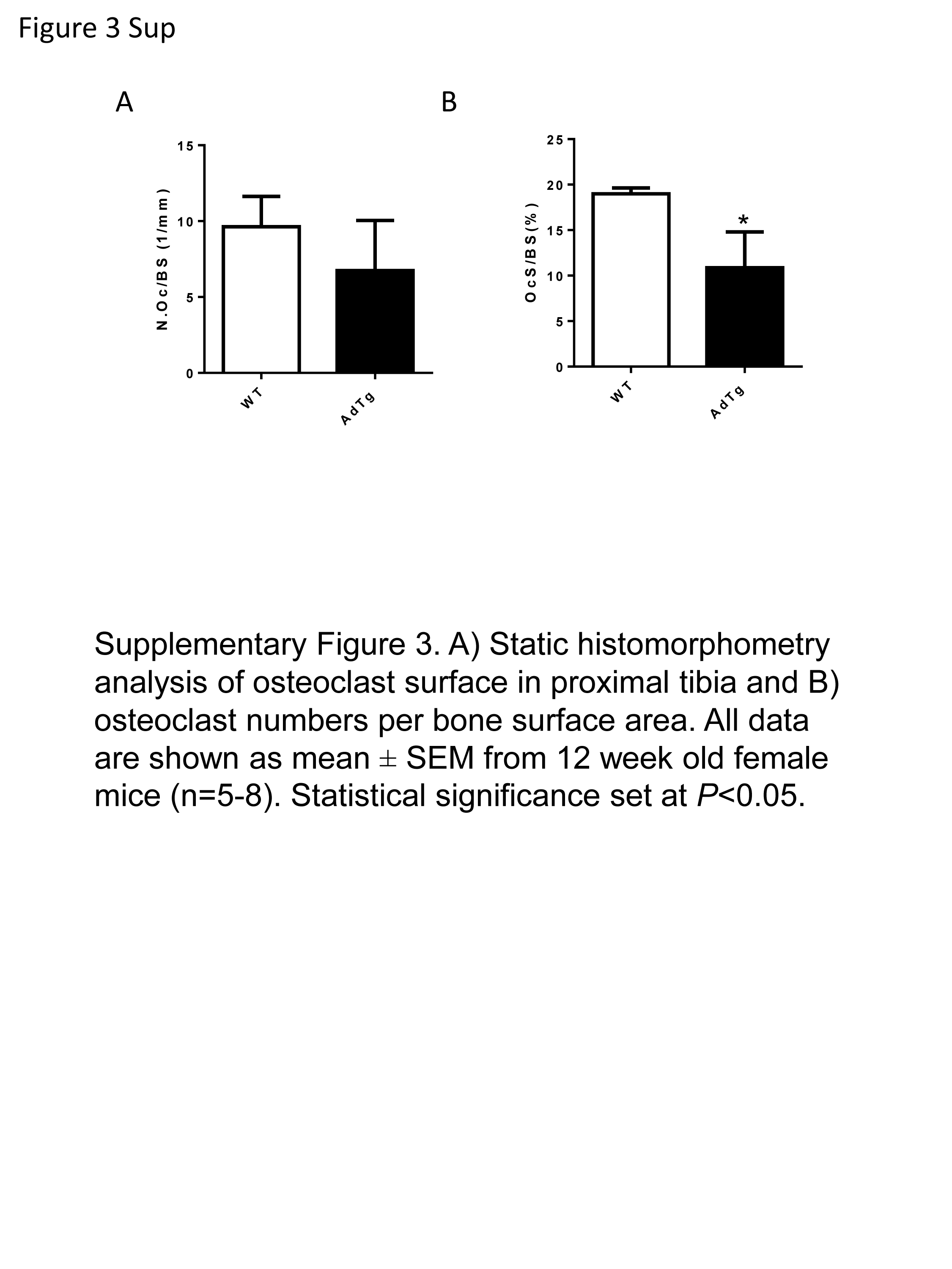

Supplement: S3 Fig — (TIF) [file pone.0134290.s003.tif]
